# Differential *In Vitro* Infection of Neural Cells by Astroviruses

**DOI:** 10.1128/mBio.01455-19

**Published:** 2019-07-09

**Authors:** Andrew B. Janowski, Robyn S. Klein, David Wang

**Affiliations:** a Department of Pediatrics, Washington University School of Medicine, St. Louis, Missouri, USA; b Department of Medicine, Washington University School of Medicine, St. Louis, Missouri, USA; c Department of Pathology and Immunology, Washington University School of Medicine, St. Louis, Missouri, USA; d Department of Neurosciences, Washington University School of Medicine, St. Louis, Missouri, USA; e Department of Molecular Microbiology, Washington University School of Medicine, St. Louis, Missouri, USA; Johns Hopkins Bloomberg School of Public Health

**Keywords:** astrovirus, astrovirus VA1, cell culture, encephalitis, virology

## Abstract

Recent advances in unbiased pathogen discovery have implicated astroviruses as pathogens of the central nervous system (CNS) of mammals, including humans. However, the capacity of astroviruses to be cultured in CNS-derived cells *in vitro* has not been reported to date. Both astrovirus VA1/HMO-C (VA1; mamastrovirus 9) and classic human astrovirus 4 (HAstV4; mamastrovirus 1) have been previously detected from cases of human encephalitis. We tested the ability of primary human neurons, primary human astrocytes, and other immortalized human nervous system cell lines (SK-N-SH, U87 MG, and SW-1088) to support infection and replication of these two astrovirus genotypes. Primary astrocytes and SK-N-SH cells supported the full viral life cycle of VA1 with a >100-fold increase in viral RNA levels during a multistep growth curve, detection of viral capsid, and a >100-fold increase in viral titer. Primary astrocytes were permissive with respect to HAstV4 infection and replication but did not yield infectious virus, suggesting abortive infection. Similarly, abortive infection of VA1 was observed in SW-1088 and U87 MG cells. Elevated expression of the chemokine CXCL10 was detected in VA1-infected primary astrocytes and SK-N-SH cells, suggesting that VA1 infection can induce a proinflammatory host response. These findings establish an *in vitro* cell culture model that is essential for investigation of the basic biology of astroviruses and their neuropathogenic potential.

## Abstract (precis)

Encephalitis remains a diagnostic conundrum in humans as over 50% of cases are managed without the identification of an etiology. Astroviruses have been detected from the central nervous system of mammals in association with disease, suggesting that this family of RNA viruses could be responsible for cases of some neurological diseases that are currently without an ascribed etiology. However, there are significant barriers to understanding astrovirus infection as the capacity of these viruses to replicate in nervous system cells *in vitro* has not been determined. We describe primary and immortalized cultured cells of the nervous system that support infection by astroviruses. These results further corroborate the role of astroviruses in causing neurological diseases and will serve as an essential model to interrogate the neuropathogenesis of astrovirus infection.

## INTRODUCTION

Inflammation of the brain, known as encephalitis, is a significant diagnostic conundrum for clinicians and researchers. While diverse numbers of viruses, bacteria, fungi, and parasites have been implicated in causing encephalitis, 30% to 63% of cases are without an identified etiology despite comprehensive testing for many of these known etiological agents (1–4). Encephalitis results in significant morbidity and mortality, as death occurs in ∼5% of patients, and in those that survive, >75% of children and >50% of adults develop long-term neurological sequelae (5–8). The recent application of unbiased next-generation sequencing to the challenge of encephalitis has identified multiple astrovirus species from cases of neurological disease in multiple mammalian species, including several cases in humans (9–16). Astroviruses comprise a family of single-stranded, positive-sense RNA viruses originally identified from stool samples of children with gastroenteritis (17, 18), with some viral particles having a distinct star-like morphological appearance as revealed by electron microscopy (19). Since 1975, multiple astrovirus genotypes have been identified from vertebrate samples, and the International Committee on Taxonomy of Viruses currently recognizes 22 astrovirus species (20). There are four species of astroviruses that have been frequently identified from human samples, including mamastrovirus 1 (representative strain human astrovirus 1), mamastrovirus 6 (representative strain MLB1), mamastrovirus 8 (representative strain VA2/HMO-A), and mamastrovirus 9 (representative strain VA1/HMO-C [VA1]) (20, 21).

While astroviruses are traditionally considered to be primary pathogens of the gastrointestinal tract, to date, nine cases of central nervous system (CNS) disease in humans have been attributed to astroviruses, including five cases of astrovirus VA1 encephalitis, two cases of astrovirus MLB2 meningoencephalitis, and single cases identified for astrovirus MLB1 and classic human astrovirus 4 (HAstV4) (9–16). Furthermore, astroviruses have been implicated in causing CNS infection in other mammals. Bovine astroviruses were detected in 34% of cases of unexplained encephalitis in cattle (22), and a novel mink astrovirus is associated with shaking mink syndrome, a neurological disease of minks characterized by tremors, ataxia, and seizures (23, 24). Astroviruses have also been detected in cases of neurological diseases in pigs and sheep (25–29). Because astroviruses were identified to have a neurotropism only recently and because of the lack of clinical diagnostic testing for astroviruses, the true burden of astrovirus-associated neurological diseases is unknown for many mammals. Given that a majority of cases of human encephalitis are without an identified etiology and the high frequency at which bovine astroviruses were detected in bovine encephalitis cases previously without an etiology, there is a distinct possibility that astroviruses could be responsible for cases of some neurological diseases in humans (1–4, 22).

The potential cellular tropisms of VA1 in the human CNS have been described previously in three cases of VA1-associated encephalitis (9–11). Astrocytes have been implicated in supporting replication in one study as VA1 capsid colocalizes with cells expressing glial fibrillary acidic protein (GFAP), a specific protein marker of astrocytes (9). In addition, VA1 capsid or RNA has also been identified in cells having a morphological appearance most similar to neurons although the neuronal lineage of VA1-positive cells was not confirmed by colocalization with any neuron-specific markers in any of the studies (9–11). In astrovirus-associated CNS diseases in other mammals, bovine and porcine astrovirus RNA or proteins have been predominantly detected in neurons (22, 28–30), while there has been only rare detection of bovine astroviruses in microglia cells (22, 30).

Although astroviruses have been propagated in multiple cell culture lines derived from non-CNS cell lineages, there are no published reports of astrovirus infection and replication in CNS cells. None of the astrovirus genotypes detected from nonhuman cases of CNS diseases have been propagated in cell culture. Among the astrovirus genotypes detected from cases of human CNS disease, growth in cell culture has been reported only for HAstV4 and VA1 (31, 32) but not in any CNS-derived cell. Therefore, we sought to develop the first CNS cell culture model of astrovirus infection using two astrovirus genotypes, VA1 and HAstV4, that have been detected in human brain tissues in cases of encephalitis (9–13, 16). On the basis of the reported localization of VA1 to astrocytes and neurons in these cases, we evaluated the capacity of VA1 and HAstV4 to productively infect primary cells from these lineages and from related immortalized cell lines.

## RESULTS

### Generation and sequencing of VA1 stock for infections.

**FIG 1:**
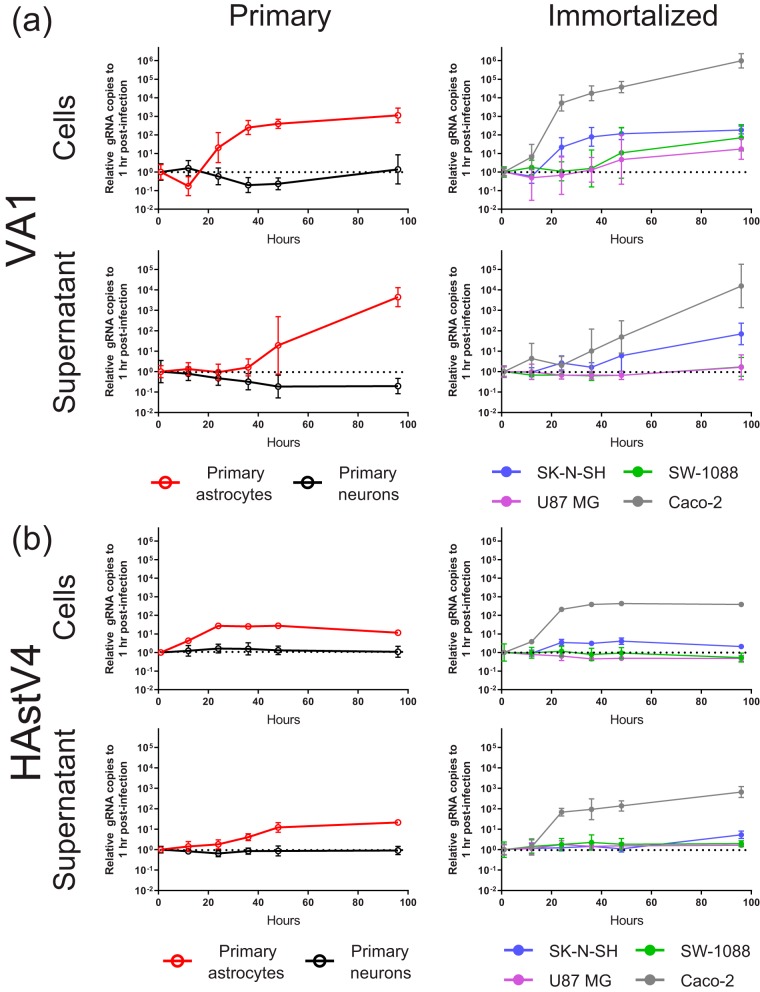
Multistep growth curves of (a) VA1 and (b) HAstV4 gRNA of primary astrocytes, primary neurons, and immortalized cell lines, including SK-N-SH, SW-1088, U87 MG, and Caco-2 cells. Each data point is normalized to the gRNA copy number present at 1 h postinoculation for each cell line. Geometric means are plotted with error bars representing 1 geometric standard deviation. The horizontal dotted line represents the relative gRNA copy number at 1 h postinoculation.

**FIG 2:**
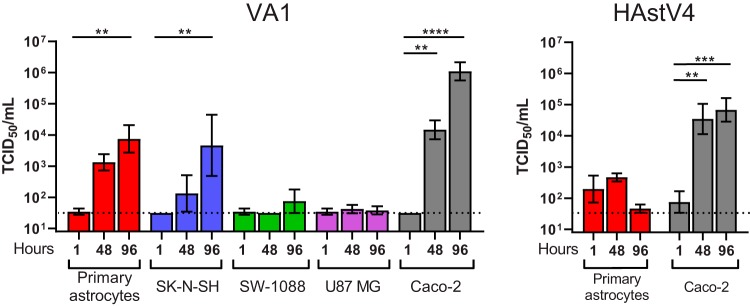
Quantification of infectious titers over time of VA1 (left panel) and HAstV4 (right panel) by a TCID_50_ assay in cell lines that had >10-fold increases in gRNA levels after inoculation. Geometric means are plotted with error bars representing 1 geometric standard deviation. The horizontal dotted line represents the limit of detection of the assay. **, *P* ≤ 0.01; ***, *P* ≤ 0.001; ****, *P* ≤ 0.0001.

We developed a high-titer VA1 viral stock by two rounds of passage in Caco-2 cells from our previously described virus stock (31). The final VA1 stock (C-P7) had a concentration of 3.16 × 10^7^ 50% tissue culture infective dose (TCID_50_) units/ml. All subsequent experiments used this stock. Whole-genome sequencing identified one additional genetic variant at nucleotide position A5470U compared to the previous stock virus (KY933670.1). This genetic variant occurs in ORF2, encoding the capsid, and resulted in a Y-to-F substitution. We confirmed replication of this VA1 stock in Caco-2 cells (Fig. 1a; see also Fig. 2), which were previously described to support replication of astroviruses (31, 32).

### Infection of primary astrocytes and neurons by VA1 and HAstV4.

In the previously described cases of VA1 encephalitis, VA1 had been detected in astrocytes and possibly in neurons of the cerebral cortex (9–11). We therefore tested the ability of primary cultures of these human cell types to support replication of VA1 and HAstV4. In primary astrocytes, an approximate 100-fold increase in the level of VA1 genomic RNA (gRNA) in the cellular fraction was detected 36 h after inoculation, with a 1,000-fold increase in the gRNA level in the supernatant fraction seen 96 h postinoculation (Fig. 1a). In addition, the infectious viral titer of VA1 increased in astrocytes over time, with a significant increase of 100-fold (*P* = 0.0083) in the viral titer at 96 h (Fig. 2). No cytopathic effect was observed in VA1-infected astrocytes.

We next determined if genetic variants of VA1 would be selected for by serial passaging the virus twice in primary astrocytes. The VA1 gRNA copy numbers in the cell lysates were similar for the two passages; passage 1 (A-P1) gave 654,450 RNA copies/ml and passage 2 (A-P2) gave 451,750 RNA copies/ml. We sequenced the VA1 genome from A-P2 and did not identify any new genetic variants relative to the starting inoculum (C-P7 stock).

We also observed an increase of over 10-fold in the levels of HAstV4 RNA in primary astrocytes in both the cellular and supernatant fractions (Fig. 1b). While we could detect an increase in the infectious titer of HAstV4 in Caco-2 cells over time, we did not detect an increase at 48 or 96 h postinoculation in primary astrocytes (for both time points, *P* = ≥0.47) or evidence of cytopathic effect (Fig. 2). These findings suggest that HAstV4 RNA replication, but not the complete life cycle, occurs in primary astrocytes.

Inoculation of primary cultures of pooled neuronal subtypes did not result in a rise in the level of gRNA for VA1 or HAstV4 or in any detectable cytopathic effect (Fig. 1a and b). We confirmed the capacity of the primary neurons to support viral replication as the Venezuelan equine encephalitis virus (VEEV) strain TC-83 had a significant increase in viral RNA over time with associated cytopathic effect (see Fig. S2 in the supplemental material). This would suggest that primary neurons are nonpermissive with respect to astrovirus infection under these experimental conditions.

10.1128/mBio.01455-19.2 FIG S2 Multistep growth curve of VEEV TC83 in primary neurons and in SW-1088 and U87 MG cells. Each data point is normalized to the PFU/viral RNA copy number ratio present at 1 h postinoculation for each cell line. The geometric mean is plotted for each data point, and geometric standard deviations are represented by error bars. The horizontal dotted line represents the PFU/RNA copy number ratio at 1 h postinoculation. Download FIG S2, EPS file, 0.1 MB. Copyright © 2019 Janowski et al. 2019 Janowski et al. This content is distributed under the terms of the Creative Commons Attribution 4.0 International license.

### Infection of immortalized cells representing the CNS.

Next, we assessed the capacity of immortalized cell lines to support replication of VA1 and HAstV4. Since primary astrocytes supported the full VA1 life cycle, we selected two immortalized cell lines from the glial lineage, namely, SW-1088 cells (derived from an astrocytoma) and U87 MG cells (derived from glioblastoma), to assess their permissiveness with respect to infection by the use of multistep growth curves. Both cell lines have been previously described to be permissive to infection by other neurotropic viruses, including Zika virus, Semliki Forest virus, and minute virus of mice (33–36). We also evaluated the capacity of SK-N-SH cells (derived from neuroblastoma), a common immortalized cell line used to study CNS viral infections (37–39). All three cell lines had increases in VA1 gRNA levels (Fig. 1a). For SK-N-SH cells, an approximate 80-fold increase in the level of VA1 gRNA in the cellular fraction was detected at 36 h postinoculation and was associated with an increase in the supernatant fraction starting 48 h after inoculation (Fig. 1a). Overall, the kinetics of the multistep growth curves for VA1 for SK-N-SH cells was similar to those for primary astrocytes. For SW-1088 and U87 MG cells, VA1 gRNA levels did not start to increase until 48 h postinoculation in the cellular fraction, with a further increase detectable at 96 h postinoculation (Fig. 1a). No increase in the level of VA1 gRNA in the supernatant fraction was observed at any time point for these two cell lines (Fig. 1a). A significant (*P* = 0.005) increase in the VA1 infectious titer was observed at 96 h in SK-N-SH cells but not in SW-1088 or U87 MG cells (Fig. 2). These results demonstrate that the full VA1 life cycle can be completed in SK-N-SH cells whereas nonproductive or abortive infection occurs in SW-1088 and U87 MG cells. As for HAstV4, we did not detect an increase in HAstV4 RNA levels in SW-1088 or U87 MG cells, while there was a small (5-fold) increase in the level of HAstV4 RNA in the cellular fraction of SK-N-SH cells (Fig. 1b). We also confirmed the capacity of SW-1088 and U87 MG cells to support viral replication, as infection with the VEEV TC-83 strain resulted in significant increases in the levels of viral RNA and of cytopathic effect in both cell lines (Fig. S2).

**FIG 3:**
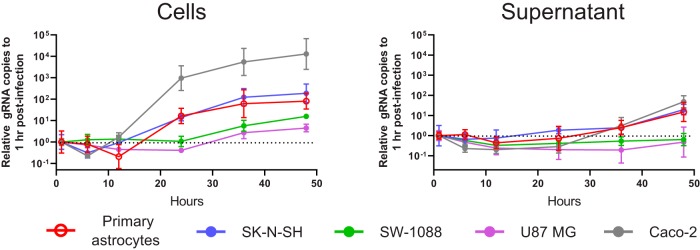
Single-step growth curve of VA1 (MOI of 3) in primary astrocytes and immortalized cell lines, including SK-N-SH, SW-1088, U87 MG, and Caco-2 cells. Data points are normalized to the gRNA copy number that was present 1 h after inoculation for each cell line. Geometric means with error bars representing 1 geometric standard deviation are plotted. The horizontal dotted line represents the relative gRNA copy number at 1 h postinoculation.

We next sought to characterize VA1 infection using a high multiplicity of infection (MOI) of 3 in the CNS cell lines, having previously demonstrated their permissiveness with respect to VA1 gRNA replication. The expected increases in VA1 gRNA levels were observed in Caco-2, primary astrocyte, SK-N-SH, SW-1088, and U87 MG cells (Fig. 3). We again observed an increase in the levels of VA1 gRNA in the supernatant of primary astrocyte, SK-N-SH, and Caco-2 cells at 48 h postinoculation.

**FIG 4:**
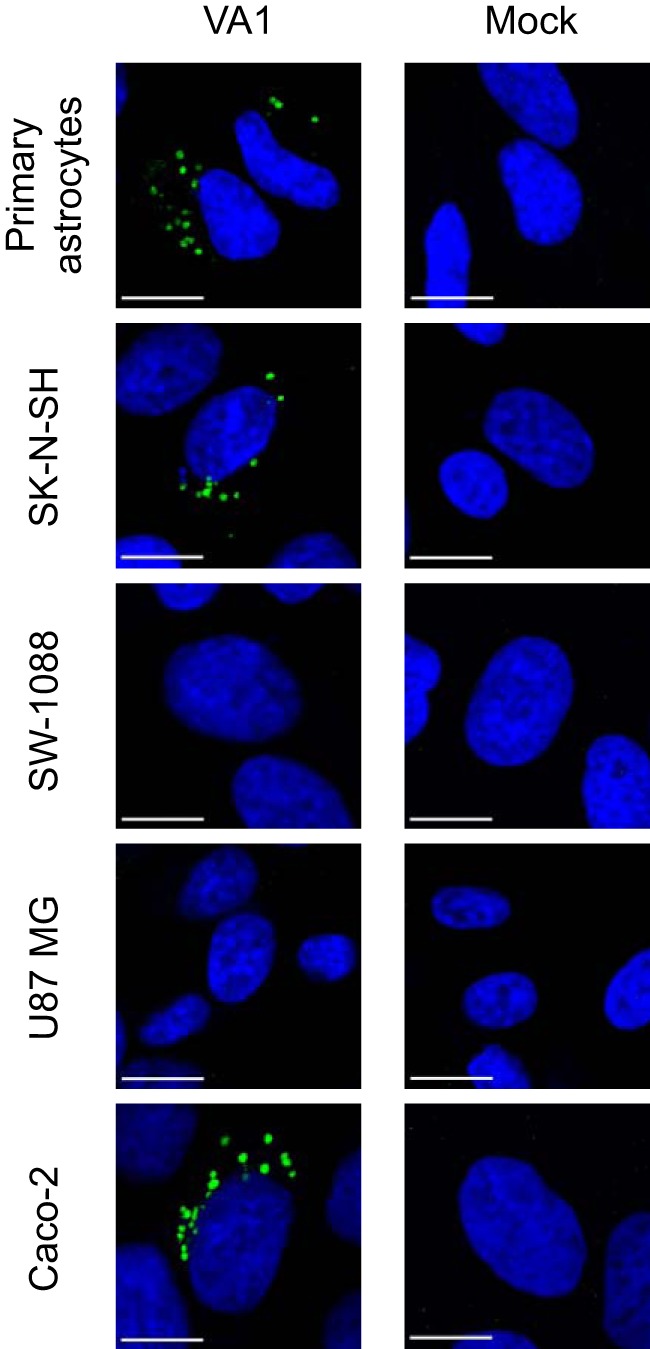
Detection of VA1 capsid in infected cells by immunofluorescence. Cells were incubated with polyclonal antibody to the capsid and labeled by staining with a secondary antibody conjugated with a green fluorescent dye. Merged fluorescence images are shown with counterstaining of nuclei (performed with DAPI). White scale bars represent 10 μm.

To evaluate viral infection at the single-cell level, we established a VA1 immunofluorescence assay (IFA) using a polyclonal antibody (Ab) to detect the VA1 capsid in cells infected at an MOI of 3. In Caco-2 cells, we detected strong punctate staining that was not observed in either mock-inoculated cells (Fig. 4) or VA1-inoculated cells stained with preimmune sera (data not shown). Similar punctate staining was also observed in primary astrocytes and SK-N-SH cells 48 h postinoculation (Fig. 4). However, we did not identify any VA1 capsid-positive SW-1088 or U87 MG cells (Fig. 4). Overall, as observed by microscopy, 0.37% of primary astrocytes, 0.81% of SK-N-SH, and 1.1% of Caco-2 cells had the characteristic punctate staining in the immunofluorescence assay.

### Characterization of abortive infection of VA1.

**FIG 5:**
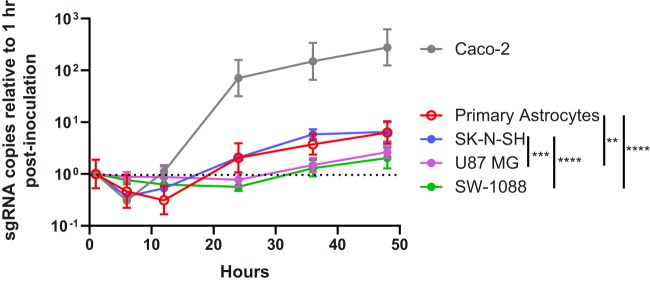
Quantification of sgRNA in single-step growth curves of VA1 (MOI of 3) from the cellular fraction of each cell line. Each plotted data point represents the geometric mean normalized to the copy number present at 1 h postinoculation with error bars representing 1 geometric standard deviation. The dotted horizontal line represents relative sgRNA present at 1 h postinoculation. The maximum *P* value for each cell line comparison at 24, 36, or 48 h postinoculation is depicted. **, *P* ≤ 0.01; ***, *P* ≤ 0.001; ****, *P* ≤ 0.0001.

During active replication, astroviruses produce a subgenomic RNA (sgRNA) strand encoding ORF2 which is translated to express the capsid protein (21, 31). Because we could not detect VA1 capsid in SW-1088 or U87 MG cells, we examined whether there was a defect in production of sgRNA in these cell types. We quantified the number of sgRNA copies by reverse transcription-quantitative PCR (qRT-PCR) and analyzed the copy number by the use of two-way analysis of variance (ANOVA) with cell type and times postinoculation (24, 36, and 48 h) as factors. There was no significant effect of the interaction between the factors [F(6, 60) = 0.6, *P* = 0.72], but cell type had a significant effect [F(3,60) = 53.1, *P* < 0.0001]. In *post hoc* analysis of each time point (24, 36, and 48 h), we detected significantly decreased quantities of sgRNA in SW-1088 or U87 MG cells compared to primary astrocytes or SK-N-SH cells (*P* = ≤0.0013 for all *post hoc* comparisons) (Fig. 5). Thus, the IFA and sgRNA results are consistent with a model where U87 and SW-1088 have limited ability to express VA1 capsid.

### Cytokine expression by nervous system cells inoculated with VA1.

**FIG 6:**
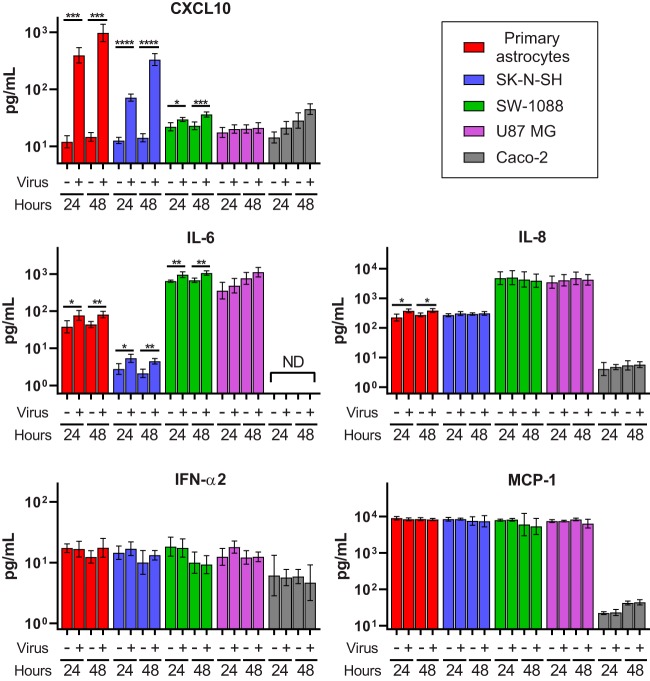
Cytokine expression in mock-infected or infected cell lines at 24 h and 48 h postinoculation. Geometric mean cytokine concentrations are plotted in picograms per milliliter with error bars representing 1 geometric standard deviation. *, *P* ≤ 0.05; **, *P* ≤ 0.01; ***, *P* ≤ 0.001; ****, *P* ≤ 0.0001.

**FIG 7:**
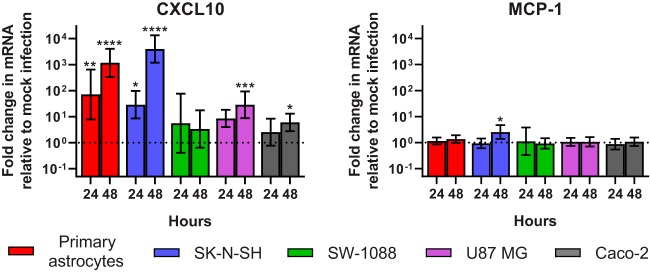
CXCL10 or MCP-1 mRNA expression in mock-infected or infected cell lines 24 h and 48 h postinoculation. Relative fold changes were calculated via the 2^−ΔΔ^*^CT^* method. Geometric means are plotted with error bars representing 1 geometric standard deviation. The horizontal dotted line represents relative baseline mRNA expression of mock-infected cells. ND, not detected. *, *P* ≤ 0.05; **, *P* ≤ 0.01; ***, *P* ≤ 0.001; ****, *P* ≤ 0.0001.

Clinically, encephalitis is characterized by infiltrates of inflammatory cells. Since VA1 did not induce cell lysis, we reasoned that infection by VA1 might induce expression of proinflammatory cytokines that would result in migration of inflammatory cells. To test this *in vitro*, we used a multiplex enzyme-linked immunosorbent assay (ELISA) to quantify the levels of the following 10 cytokines in the supernatant: interferon alpha 2 (IFN-α2), interferon gamma (IFN-γ), tumor necrosis factor alpha (TNF-α), interleukin-1α (IL-1α), IL-1β, monocyte chemoattractant protein 1 (MCP-1), CXCL10/IP-10, IL-6, IL-8, and vascular endothelial growth factor (VEGF). We identified 5-fold to 60-fold increases in CXCL10 levels in the supernatant of VA1-infected primary astrocytes or SK-N-SH cells (Fig. 6). We also detected small changes in IL-8 from infected primary astrocytes and IL-6 in infected primary astrocytes, SK-N-SH, and SW-1088 cells (Fig. 6). There was no change in the level of expression of MCP-1 or IFN-α2 in infected cells (Fig. 6). VEGF and IFN-γ also did not have altered expression upon infection, and many samples did not have detectable amounts of IFN-γ, TNF-α, IL-1α, or IL-1β (data not shown). We further corroborated the results of the CXCL10 and MCP-1 ELISA by qRT-PCR of host mRNA. There were significant increases in the numbers of CXCL10 transcripts in primary astrocytes and SK-N-SH cells, but MCP-1 expression did not significantly change upon infection in multiple cell lines except 48 h postinoculation in SK-N-SH cells (Fig. 7).

## DISCUSSION

The recent detection of astroviruses from human and other mammalian brain tissue has identified this family of viruses as newly recognized neuropathogens. However, to date, no astrovirus has been cultivated in any CNS cell line. Here, we describe the ability of both primary astrocytes and SK-N-SH cells to support the complete life cycle of VA1, as evidenced by increasing levels of viral gRNA and sgRNA over time, detection of intracellular viral capsid, and increasing infectious viral titers over time. The development of a CNS cell culture system is integral to understanding the neuropathogenesis of VA1 and other mammalian astroviruses that have been associated with neurological diseases. Furthermore, we identified additional cell types that appear to be permissive with respect to both VA1 and HAstV4 infection and replication; however, these cells are unable to complete the viral life cycle, as no increase in the infectious titer could be detected. Nonetheless, these cell lines can also make valuable contributions to understanding of viral pathogenesis, as for some virus-mediated CNS diseases, such as those involving rabies virus or La Crosse virus, abortive infection is an important component of disease pathogenesis (40–42).

The isolate of VA1 used in this study was derived from a stool sample from the original outbreak of VA1-associated gastroenteritis (43). There are 3.4% to 4.6% nucleotide and 1 to 3.2% amino acid polymorphisms between the four published genome sequences of VA1 from encephalitis cases and the reference stool case (9–12, 43). From the molecular epidemiologic data alone, it was not clear whether all VA1 viruses have neurotropic potential or whether specific viral variants or adaptations are required for neurotropism. Our data suggest that genotypes of VA1 found in human stool have the ability to replicate in CNS cells *in vitro*. Humans are frequently exposed to VA1, as seroprevalence is 65% in adulthood, but it is unclear what subset of exposures leads to encephalitis (44). All five VA1 encephalitis cases occurred in immunocompromised patients who either had X-linked agammaglobulinemia or had recently received a hematopoietic stem cell transplant, suggesting that host immunity may play an important role in further modulating the risk of developing encephalitis. This risk could be further potentiated by the different genotypes of VA1, as the genotypes from the cases of VA1-associated encephalitis could contain genetic variants that confer even greater CNS pathogenicity. We assessed whether there was high evolutionary pressure on the stool-derived VA1 genome with two serial passages in primary astrocytes, but we did not detect any new genetic variants in the viral genome. Further long-term passaging experiments might identify genetic variants that do confer greater replicative capacity in astrocytes. The effect on replication of the currently known genetic variants between the stool-derived and brain-derived VA1 genotypes could be tested in the future with development of a reverse genetics system.

In one of the VA1-associated encephalitis cases, VA1 capsid was localized to astrocytes by immunohistochemistry performed with costaining for GFAP (9). Consistent with this finding, we demonstrated that primary astrocytes are fully permissive for VA1 infection. In three of the cases of VA1-associated encephalitis, VA1 RNA or capsid was detected in cells with a morphology most consistent with neurons. However, we did not detect any increase in astrovirus gRNA levels in primary neurons. It is formally possible that the cells described in the published cases of encephalitis were not neurons as neuron-specific markers were not used to colocalize the virus. Alternatively, the primary neuronal culture system that we used may not fully mimic the *in vivo* milieu of the human brain that is required for astrovirus infection. Furthermore, there may be specific neurons derived from specific lineages or locations within the brain that are permissive with respect to astrovirus infection that are not included in the pooled neuronal cultures. We did identify completion of the full life cycle in SK-N-SH cells, an immortalized neuroblastoma cell line commonly used to model CNS viral infections. This cell line could provide future insights into particular CNS lineages or states of differentiation that support VA1 infection, as SK-N-SH cells can be induced to differentiate by retinoic acid (45).

When SW-1088 and U87 MG cells were inoculated with VA1, an increase in the level of VA1 gRNA was observed, but this was associated with delayed production of sgRNA and lack of detection of VA1 capsid or infectious viral particles. Thus, these cells appear to be permissive with respect to virus entry and replication but are incapable of completing the entire viral life cycle. It is possible that these cells lack expression of an essential proviral gene or, alternatively, that they might express an inhibitory factor that prevents completion of the full VA1 life cycle. U87 MG and SW-1088 cells have elevated production of IL-6 and IL-8 in comparison to cell lines that support the full viral life cycle, as these cytokines contribute to their oncological pathogenicity (46, 47). IL-6 can potentiate the antiviral effect of interferon alpha, and exogenous IL-8 can inhibit HIV replication, raising the possibility that these cytokines could confer additional antiviral effects that contribute to abortive VA1 infection (48, 49).

CXCL10 expression was significantly increased upon infection of VA1 in primary astrocytes and SK-N-SH cells. CXCL10 is an important chemoattractant induced by many other viruses upon infection, resulting in recruitment of immune cells (50). In addition, CXCL10 has been noted to induce neuronal apoptosis, contributing to viral pathogenicity upon infection, including infection by West Nile virus (51–53). Infiltrates of lymphocytes and loss of neurons were identified in the histological examinations of the cases of VA1 encephalitis (9–11), and CXCL10 could be an important mediator of this observed inflammatory response to infection. Further development of an *in vivo* model of VA1 infection would better characterize the role of this cytokine upon VA1 infection.

Abortive infection with RNA replication has not been previously reported for any classic human astrovirus in a CNS cell line. In the published case report of HAstV4 encephalitis, brain tissue was positive for viral RNA by PCR but viral capsid was not detectable by immunohistochemistry, consistent with our model of abortive infection (16). These findings would suggest that there are differences between the host-virus interactions of HAstV4 and astrocytes and those of VA1 and astrocytes. Our *in vitro* observations suggest that VA1 has a greater capacity to cause CNS infections. We previously described one key distinction, as, in contrast to the classic human astroviruses, VA1 does not require trypsin for propagation in cell culture (54). One possibility is that for HAstV4, nervous system cells may require trypsinization conditions different from those that enable productive infection of Caco-2 cells. It is also possible that astrocytes are inherently capable of supporting only abortive infection of HAstV4. Alternatively, our HAstV4 genotype may not be well adapted for infection of the CNS as it was originally isolated from stool. Abortive infection may still be relevant to the host, as an inflammatory response could be induced in the absence of the completion of the full viral life cycle. While we did detect small increases in the levels of CXCL10 and IL-6 with abortive infection of VA1 in SW-1088 cells, it is unclear whether these increases are biologically significant, and further characterization may reveal host responses that are significantly induced by abortive infection.

In summary, we have developed the first cell culture system of astrovirus infection in nervous system cells. This is another important step in demonstrating the pathogenicity of astroviruses in causing neurological diseases, and we can now begin to explore the essential viral and host factors that contribute to CNS disease. Growth of a virus in pure culture fulfills one of Koch’s postulates. If an *in vivo* model of CNS infection by astrovirus VA1 could be developed, this would be the final step in fulfilling Koch’s postulates and demonstrate that astrovirus VA1 is a bona fide pathogen of the CNS.

## MATERIALS AND METHODS

### Cell culture.

All cells were maintained at 37°C with 5% CO_2_. Caco-2, SW-1088 (HTB-12; ATCC), SK-N-SH (HTB-11; ATCC), and U87 MG cells were maintained in Dulbecco’s modified Eagle media (DMEM) with l-glutamine (Gibco) supplemented with 10% fetal bovine serum (FBS; Gibco) and 1% of 10,000 units/ml of penicillin/streptomycin (Gibco).

Primary human astrocytes from the cerebral cortex were commercially purchased and maintained in astrocyte media with 2% FBS, astrocyte growth supplement, and penicillin/streptomycin (Sciencell). Cells were seeded on poly-l-lysine (Sigma)-treated tissue culture plasticware at 2 μg/cm^2^. For all experiments, the passage number for astrocytes was less than or equal to two. Primary neurons pooled from several brain regions were also commercially purchased and maintained in neuronal media supplemented with neuronal growth supplement and penicillin/streptomycin (Sciencell). Neurons were maintained on poly-l-lysine-coated plasticware (2 μg/cm^2^) and allowed to incubate 10 to 14 days for neurite maturation prior to any experimentation.

### Virus infection protocols.

VA1 infections were performed as previously described (31, 55), and the same conditions were used for VEEV TC83 infections. For infections performed with HAstV4, an aliquot of the virus was activated with 100 μg/ml of trypsin for 30 min (56). VA1 or VEEV aliquots were not pretreated with trypsin. VA1, HAstV4, or VEEV aliquots were diluted in DMEM or in astrocyte/neuronal media without FBS. Cells were washed once with serum-free media (SFM), incubated with the virus mixture for 1 h, and washed once with SFM. For cells incubated with VA1 or VEEV, growth media with FBS was added to the cells. For HAstV4, SFM supplemented with 3.3 μg/ml of trypsin was added to the cells except for SK-N-SH and primary neurons. In pilot studies, we identified death of SK-N-SH cells maintained in serum-free media. Significant detachment also occurred in SK-N-SH cells and primary neurons treated with trypsin. Therefore, HAstV4-inoculated SK-N-SH cells were maintained in media containing FBS without trypsin, and no trypsin was added to the growth media of HAstV4-inoculated neurons.

### Generation of viral stocks.

Using a previously published VA1 stock (C-P5) (31), Caco-2 cells grown in T175 flasks were infected with C-P5 at an MOI of 0.006 and incubated for 7 days to generate a new stock (C-P6) (55). The flasks were freeze-thawed three times to generate whole-cell/supernatant lysate. Next, C-P6 was inoculated into T175 flasks containing confluent Caco-2 cells at an MOI of 0.01 and incubated for 5 days. Flasks were freeze-thawed three times, and the lysate (C-P7) was quantified by a TCID_50_ assay (3.16 × 10^7^ TCID_50_ units/ml) and used in all subsequent experiments (55).

A stock of HAstV4 was also generated by passage in Caco-2 cells. A 100-μl aliquot of stock virus was activated by incubation with 100 μg/ml of trypsin (Gibco) for 30 min. The trypsin-treated virus was then inoculated into Caco-2 cells, and the cells were maintained in SFM supplemented with 3.3 μg/ml of trypsin for 7 days. The cells were freeze-thawed three times, and the viral titer was determined by a TCID_50_ assay (2.8 × 10^5^ TCID_50_ units/ml). This stock was used in all HAstV4 experiments.

C-P7 VA1 was passaged twice in primary astrocytes grown in a 6-well plate, starting with an initial MOI of 0.1. After 5 days, the cells were freeze-thawed three times (A-P1 stock), and 200 μl of cell lysate was added to uninfected astrocytes. The cells were incubated for 5 days and freeze-thawed three times (A-P2 stock), and RNA was extracted from the cell lysate of both passages.

### Sequencing the VA1 genome.

TRIzol reagent (Thermo Fisher) was added to 100 μl of the C-P7 stock or 200 μl of the A-P2 stock, and RNA was extracted using Direct-zol miniprep columns (Zymo Research). Reverse transcription was performed using oligo(dT) with Superscript IV (Thermo Fisher) at a RT temperature of 60°C for 10 min. The cDNA was then used in PCRs with PfuUltra II Fusion HS DNA polymerase (Agilent) and with previously published primer combinations for sequencing of the VA1 genome (31). For sequencing of the VA1 isolate from A-P2, two changes were made to the PCR protocol. First, the number of PCR cycles was increased to 40 for all primer combinations of the genome. Second, the annealing temperature for fragments 1 and 4 was increased to 58°C. The genome from each stock was sequenced in three independent experiments, and genetic variants were identified if they were present in at least two of the three experiments.

### Single and multistep growth curves.

For multistep growth curve analyses, VA1 or HAstV4 was inoculated into the cell lines at an MOI of 0.01 with three replicates for each time point. For VEEV, an MOI of 0.1 was used. Cell and supernatant fractions were collected at 1, 12, 24, 36, 48, and 96 h after inoculation. In single-step growth curves for VA1, cells were infected at an MOI of 3 with three replicates at each time point. Both cell and supernatant fractions were collected at 1, 6, 12, 24, 36, and 48 h after inoculation. The supernatant was collected for both growth curves and frozen at −80°C. The cellular fraction was collected by adding TRIzol reagent to the wells, transferring the mixture to microcentrifuge tubes, and freezing at −80°C.

### RNA extraction and qRT-PCR.

Aliquots of the supernatant fraction were lysed in TRIzol. RNA from the cellular or supernatant fractions was extracted by the use of Direct-zol 96-well plates (Zymo Research). VA1 gRNA was quantified using a previously published TaqMan-based qRT-PCR assay (31, 55). To quantify HAstV4 RNA, we adapted a previously published qRT-PCR assay (16, 57). The HAstV primers and probe were as follows: forward primer, 5′-TCAACGTGTCCGTAAMATTGTCA-3′; HAstV reverse primer, 5′-TGCWGGTTTTGGTCCTGTGA-3′; TaqMan probe, 5′-(56–6-carboxyfluorescein [FAM])CAACTCAGG/ZEN/AAACARG/3IABkFQ/-3′ (Integrated DNA Technologies [IDT]). For quantification of VA1 sgRNA, we developed a qRT-PCR assay targeting a region within ORF2 common to both the genomic and subgenomic strands (sg/gRNA qRT-PCR assay) using the following primers and probe: forward primer AJ135 (5′-GTGTTGGACCAAGATCAGATG-3′), reverse primer AJ137 (5′-CAGAACTAGAGGAGTCTGAATCC-3′), and probe AJ136 (5′-56-FAM/TCAAATTCA/ZEN/GCATCGCTACAGATTGACC/3IABkFQ/-3′) (IDT). VEEV RNA was quantified by the use of a previously published qRT-PCR protocol and normalized to the calculated PFU (58).

To generate positive-control RNA, we used two plasmids as the templates for *in vitro* transcription (IVT). Plasmid DW737 contains a 370-bp sequence of the HAstV4 genome, and plasmid DW738 contains a 490-bp region containing the VA1 region for the sg/gRNA qRT-PCR. Both plasmids were linearized with Pst-I (New England Biolabs) and subjected to RNA transcription using a MEGAscript T7 kit (Thermo Fisher). The RNA was purified using an RNeasy kit (Qiagen) and quantified using a NanoDrop 1000 spectrophotometer (Thermo Scientific). qRT-PCR was performed using TaqMan Fast Virus 1-Step master mix (Applied Biosystems) and a ViiA 7 real-time PCR system (Applied Biosystems), with an annealing temperature of 55°C. Three replicates of 10-fold serial dilutions of the IVT RNA were used to generate a standard curve. The cycle threshold (*C_T_*) and RNA copy number data were plotted, and a linear regression model was determined from the values using Prism version 8.0.2 (GraphPad) to quantify viral RNA (see Fig. S1 in the supplemental material).

10.1128/mBio.01455-19.1 FIG S1 qRT-PCR standard curves for quantification of HAstV4 RNA copies and VA1 sg/gRNA copies. Serial 10-fold dilutions of HAstV4/VA1 *in vitro*-transcribed RNA were used as control templates. A line of best fit and a coefficient of determination were calculated. Error bars represent one standard deviation. Download FIG S1, EPS file, 0.02 MB. Copyright © 2019 Janowski et al. 2019 Janowski et al. This content is distributed under the terms of the Creative Commons Attribution 4.0 International license.

The copy number for VA1 or HAstV4 RNA was calculated for each time point for the single-step and multistep growth curves from two separate experiments. Data from samples with undetectable levels were transformed to the *C_T_* limit of detection for each assay. These values were normalized to the 1-h time point, and the geometric mean and geometric standard deviation were calculated for each time point and graphed in Prism.

For calculation of VA1 sgRNA, we subtracted the value corresponding to the result of the gRNA assay from that corresponding to the sg/gRNA assay result. This value was normalized to the 1-h time point with geometric means and geometric standard deviations calculated and graphed in Prism. We compared logarithmically transformed data from primary astrocytes and SK-N-SH, SW-1088, and U87 MG cells at time points 24, 36, and 48 h using a two-way ANOVA with a *post hoc* analysis of multiple comparisons completed using Tukey’s multiple-comparison test in Prism. Adjusted *P* values of ≤0.05 were considered significant.

For quantification of host mRNA, extracted RNA from mock-infected cells and infected cells (MOI of 3) at 24 and 48 h postinoculation was treated with DNase I (Thermo Fisher) for 15 min at room temperature. EDTA was then added, and the mixture was incubated at 65°C for 10 min. TaqMan Fast Virus 1-Step master mix was then added to the DNase-treated samples, and the reaction mixture was analyzed by qRT-PCR using commercial assays for the following: RPLP0 (IDT; catalog no. Hs.PT.39a.22214824), MCP-1 (IDT; catalog no. Hs.PT.58.45467977), and CXCL10 (IDT; catalog no. Hs.PT.58.3790956.g). The annealing temperature for all assays was 55°C. Relative quantities of RNA were calculated using the 2^-ΔΔ^*^CT^* method, with RPLP0 as the housekeeping gene, and graphed via Prism. Undetectable samples were normalized to a *C_T_* value of 40. The Δ*C_T_* value for each gene was also calculated for mock-infected or infected cells at each time point and compared via multiple *t* tests with a correction for multiple comparisons by the Holm-Sidak method in Prism. Adjusted *P* values of ≤0.05 were considered significant.

### Quantification of infectious particles by TCID_50_ assays.

We used a previously published qRT-PCR-based TCID_50_ assay to quantify VA1 or HAstV4 infectious particles (31, 55). All viral infections were performed in triplicate with completion of two independent experiments. Whole-cell lysates were collected 1, 48, or 96 h postinoculation and freeze-thawed three times. Lysates from HAstV4-infected cells were incubated with 100 μg/ml of trypsin for 30 min, while the VA1 cell lysate was not pretreated with trypsin. Caco-2 cells grown in 96-well plates were then infected using 10-fold dilutions of each lysate in quadruplicate, starting with 10 μl of the cell lysate per well. After the infection step, Caco-2 cells inoculated with HAstV4 were maintained in SFM supplemented with 3.3 μg/ml of trypsin, while VA1-infected cells were maintained in FBS-containing media. Cells were incubated for 72 h and cell fractions collected in TRIzol for RNA extraction. qRT-PCR was performed on the RNA extracts using the HAstV4 or VA1 assays. The Spearman-Karber method was used to calculate the TCID_50_ value for each sample as wells were deemed infected if the *C_T_* value from the qRT-PCR assay was <30 (31, 55, 59, 60). The geometric mean for each viral titer at each time point was calculated, and the data from the different time points were compared using a Kruskal-Wallis test with *post hoc* testing by Dunn’s multiple-comparison test; *P* values of ≤0.05 were considered significant.

### Production of rabbit polyclonal anti-VA1 capsid antibody.

The VA1 capsid sequence was analyzed using OptimumAntigen (GenScript) to identify antigenic regions, and an optimal peptide sequence was selected from amino acid positions 532 to 545 (YP_003090288.1) for antibody production. A cysteine residue (CNSEEWHTNAEQPHQ) was added to the N terminus to facilitate conjugation. The peptide was commercially produced and inoculated into rabbits by the vendor (GenScript). Preimmune sera and affinity-purified antibody (WAB111; 1.32 mg/ml) were collected from sera 35 days postinoculation.

### Immunofluorescence assay for detection of VA1 capsid in cell culture.

Glass coverslips were coated with sterile 0.1% gelatin or 2 μg/cm^2^ of poly-l-lysine (primary astrocytes) for at least 1 h in a 24-well plate. The mixture was removed, and cells were added to the wells. After adhering to the plate for 24 to 48 h, cells were either subjected to mock infection or infected with VA1 at an MOI of 3. At 48 h postinoculation, cells were washed once with phosphate-buffered saline (PBS) and fixed with 4% paraformaldehyde for 10 min. The cells were washed 3 times with PBS and then permeabilized with PBS with 0.1% Triton X-100 for 10 min. The cells were washed once with PBS for 5 min and then blocked overnight with 10% horse serum–PBS at 4°C. After blocking, a 1:1,000 dilution of WAB111 or preimmune sera in PBS with 10% bovine serum albumin and 0.05% Tween 20 (Ab buffer) was added to the coverslips. After a 1 h incubation at 4°C, the coverslips were washed for 5 min with PBS–0.05% Tween 20 (PBS-T) three times. A 1:1,000 dilution of donkey anti-rabbit antibodies conjugated with Alexa Fluor 488 (Thermo Fisher) was diluted in Ab buffer and incubated with the coverslips at room temperature for 1 h. The cells were washed five times with PBS-T and stained with 600 nM DAPI (4′,6-diamidino-2-phenylindole; Thermo Fisher) for 5 min, and a final wash with PBS-T was performed. Coverslips were mounted using ProLong Gold Antifade mounting media (Thermo Fisher). The coverslips were then visualized using a Zeiss LSM880 confocal laser scanning microscope at ×63 magnification. Images were cropped and labeled using the Zen 2.3 lite application (Carl Zeiss Microscopy). For quantification of the frequency of infected cells, a 1.1-mm-by-1.1-mm region from each coverslip was imaged, with a total of two coverslips imaged for each cell type. The number of VA1-positive cells was counted and divided by the total number of cells in the field as represented by the number of DAPI-positive nuclei determined by the use of Fiji software.

### Cytokine expression of infected cells.

Supernatant fractions of two independent infection experiments (MOI of 3; 3 replicates each) were analyzed by multiplex ELISA for IFN-α2, IFN-γ, TNF-α, IL-1α, IL-1β, MCP-1, CXCL10, IL-6, IL-8, and VEGF (Milliplex; MilliporeSigma). Duplicate 25-μl volumes of each sample were added to a 96-well plate and diluted in assay buffer with premixed beads targeting each individual cytokine. Samples were incubated overnight at 4°C on a plate shaker. The plate was then washed twice, and detection antibodies were added. The plate was incubated for 1 h at room temperature, and then streptavidin and phycoerythrin were added to each well. The plate was incubated for 30 min at room temperature and then washed twice. The plate was then analyzed on a Bio-Plex 200 system (Bio Rad). Geometric mean concentrations were calculated for each cytokine, and differences between mock-infected and infected samples were determined by multiple *t* tests with correction for multiple comparisons by the use of the Holm-Sidak method in Prism. Adjusted *P* values of ≤0.05 were considered significant.
